# Time-domain analysis for extracting fast-paced pupil responses

**DOI:** 10.1038/srep41484

**Published:** 2017-01-30

**Authors:** Alexandre Zénon

**Affiliations:** 1 Institute of Neuroscience, Université catholique de Louvain, Brussels, Belgium, INCIA, Université de Bordeaux, CNRS UMR5287, 33076 Bordeaux, France

## Abstract

The eye pupil reacts to cognitive processes, but its analysis is challenging when luminance varies or when stimulation is fast-paced. Current approaches relying on deconvolution techniques do not account for the strong low-frequency spontaneous changes in pupil size or the large interindividual variability in the shape of the responses. Here a system identification framework is proposed in which the pupil responses to different parameters are extracted by means of an autoregressive model with exogenous inputs. In an example application of this technique, pupil size was shown to respond to the luminance and arousal scores of affective pictures presented in rapid succession. This result was significant in each subject (*N* = 5), but the pupil response varied between individuals both in amplitude and latency, highlighting the need for determining impulse responses subjectwise. The same method was also used to account for pupil size variations caused by respiration, illustrating the possibility to model the relation between pupil size and other continuous signals. In conclusion, this new framework for the analysis of pupil size data allows us to dissociate the response of the eye pupil from intermingled sources of influence and can be used to study the relation between pupil size and other physiological signals.

The response of the pupil to light intensity variations has been known for a very long time and used as a diagnostic tool in medicine. Photons activate the cones, the rods and the ganglion cells in the retina^1^, which then send projections to the pretectal olivary nucleus, whose signal is relayed to the Edinger-Westphal nucleus^2^. Preganglionic parasympathetic neurons then project to the ciliary ganglia, which control the constriction of the pupils by innervating the pupillary sphincters. However, in addition to this well-known circuit, pupil size is also affected by other sources of influence, whose origins are less clear. Lesions of the pretectal olivary nucleus abolish the light reflex, but leave intact the response of the pupil to colour and grating stimuli^3^. Complex visual information such as the presence of the image of the sun^4^, and covert attention^5^ affect the pupil response. Cognitive tasks, such as mental calculation or working memory load also lead to very robust increases in pupil size^6^. Similarly, pupil size responds to physical effort^7^, violation of predictions^8^,^9^,^10^, shifts in the exploration/exploitation tradeoffs^11^, etc. The neural origin of these various sources of influence on pupil size remains an intense topic of study^12^,^13^ but is likely to involve the arousal system, including Locus Coeruleus and basal forebrain^12^,^14^,^15^,^16^.

From a methodological point of view, a common issue with the analysis of the pupil size data is the slowness of the pupil size variations, which impedes the analysis of the pupil response to fast-paced events. In a previous work addressing these issues, Hoeks and Levelt have proposed the use of a deconvolution technique to analyze the pupil size data^17^. In this method, the pupil size data is deconvolved with a canonical impulse response. Even though the method was initially used on very simple designs, it was later adapted and applied successfully to more complex paradigms^18^,^19^. However, this method is limited in several ways. First and foremost, it does not take care of the very strong low-frequency noise found in pupillometric data. Baseline subtraction - i.e. subtracting the mean of the pupil size data taken from a portion of time preceding the event onset - is the most common method used to get rid of this noise but is of limited efficacy because it does not deal with the slope of the signal at the event onset. As a consequence, this method requires many trial repetitions in order for the noise to cancel out. Another issue with the deconvolution method for pupillometric data analysis relates to the high between-subject variability in the shape of the impulse response and in the delay between the event onset and the beginning of the response (as detailed below). Since the method relies on the use of a common canonical impulse response, this variability is not taken into account.

Here I propose to use a system identification framework to optimize the analysis of pupillometric data (see ref. 20 or refs 21, 22, 23 for applications of similar methods to neuroimaging and electroencephalographic data, respectively). In particular, the low-frequency noise is modeled with an autoregressive model, in which each data point is predicted by the value of the data points that precede it, within a given time window. In addition, the pupillary response to specific events is modeled by adding exogenous inputs. I apply this approach in a passive task in which participants are presented with fast-paced affective pictures standardized in their arousal content, and in a rest recording session during which respiration is recorded concurrently. It is noteworthy that the objective of the present work is not to provide a biologically plausible model of pupil responses, which has been a very active topic of study in the context of the light reflex^24^,^25^, but only to propose a new approach to analyze the pupil signal in psychophysiological experiments. The scope of the present study is not either to propose an exhaustive validation of the technique with extended dataset, but simply to introduce its concept and illustrate it in two example applications.

## Methods

### Participants, procedure and tasks

Five healthy subjects (1 female, age range 23–38) were recruited to participate in the present study after signing an informed consent form. All experimental procedures were approved by the local ethics committee (Comité d’Ethique Hospitalo-Facultaire, Saint-Luc hospital, Brussels, Belgium) and were in accordance with the Helsinki declaration.

Subjects were seated in front of a 19” CRT screen cadenced at 100 Hz, with their head resting on a chinrest. An Eyelink 1000 eye tracker was situated just below the screen, and centered on the dominant eye of the participant. The eye tracker recorded the diameter of the pupil and the gaze position at 500 Hz. Given that the absolute dimension of the recorded pupil is irrelevant to the present work, the diameter of the pupil was not calibrated and is therefore reported in arbitrary units.

Five participants participated in a *passive image-viewing* task, while one participant also underwent a *rest recording* session. In the *passive image-viewing* task, participants were presented with a series of 1055 images selected from the NAPS bank (i.e. all images with landscape orientation; see ref. 26 for details on the properties of the images). Each image was 19.3 degrees wide, 14.5 degrees high and was shown for 200, 300 or 400 ms. The presentation duration was randomized with a uniform distribution and the order of the images was pseudorandomized. There were 4 sub-blocks of image presentation including 263 or 264 images each, and separated by a fixation screen lasting 6 to 7 seconds. A similar fixation screen began and ended the image-viewing task. Within image presentation sub-blocks, there was no delay between successive image presentations. Participants were instructed to maintain their gaze on a central white fixation cross, superimposed on the images and to minimize the number of blinks executed during the tasks. The total duration of the task was 5′45”. Participants performed 2 blocks of this task: in the non-scrambled block, the images from the NAPS bank were not altered, whereas in the scrambled block, the images were phase scrambled^27^.

One participant underwent also the *rest recording* session, during which he had only to fixate a central fixation cross on the computer screen and to minimize blinks. This experiment was merely a proof of concept for the possibility of using ARX for linking physiological signals to pupil size data, which is why only one participant was included. An inflatable cuff was attached around participant’s waist and connected to a pressure transducer, which converted the pressure measured in the cuff into voltage. This signal was recorded at 100 Hz with a 16-bit data acquisition card (National Instruments, Austin, Texas, USA) connected to the computer running Matlab (data acquisition toolbox, The MathWorks, Inc., Natick, Massachusetts, USA). This recording session lasted 2 minutes.

### Pupil signal pre-processing

Blinks were tagged by the Eyelink blink detection algorithm. 100 ms of data were additionally tagged as blinks right before and after each automatically detected blink epochs, to ensure that no artifacts were left in the data. Blinks were then linearly interpolated. The pupil size data was downsampled to 10 Hz by taking the mean of the pupil size in successive 100 ms bins. Different downsampling frequencies were tested and higher sampling rate did not improve the results.

All analyses were performed with Matlab and the system identification toolbox (The MathWorks, Inc., Natick, Massachusetts, USA). Since we were interested solely in the pupil responses to the stimulation and not in the baseline pupil size, the DC component of the signal was irrelevant and the mean was subtracted from the overall signal prior to time series analyses. All the functions used in the present paper are provided as a toolbox on a public repository: https://github.com/alexandre-zenon/pupil.

### ARX model

The autoregressive model with exogenous inputs (ARX) was as follows:


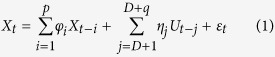


where *X*_*t*_ is the pupil signal at time t, *U*_*t*_ is the input matrix at time t, *φ* is the autoregressive parameter vector of length *p, η* is the input parameter vector of length *D* + *q (D* standing for delay), whose values between 0 and *D* are all zero, and *ε*_*t*_ is the noise at time t. *ε* is modeled as a gaussian noise with mean zero and variance *σ*, which is also an unknown parameter of the model. The inputs varied according to the analyses and are described below in the results section.

The orders (*p,q*) and delay (*D*) of the model were chosen so as to minimize the Akaike Information Criterion (AIC) and were fitted by minimizing the square error of the *t* + *1* prediction (maximum likelihood estimation, arx function in matlab), while ensuring stability (i.e. the roots of the polynomial expression of the ARX model remained within the unit circle). The same analysis was also conducted with the Scwharz criterion instead of the AIC (Lütkepohl 1985). This typically resulted in larger model orders but did not affect the main outcome of the analysis (significant arousal response to image presentation in all participants, which was absent when using scrambled images).

One potential difficulty in using the ARX approach in practical applications is in the building of the design matrix. Similarly to general linear models, used for example in the context of functional neuroimaging analysis^28^, the matrix has to be full rank, which means that no column can be linear combinations of the other columns. Likewise, the columns of the matrix should not correlate too much with each other. In the present case, because the image presentation was divided in 4 sub-blocks, separated by 7-second breaks, all the vectors representing the image features correlated strongly with each other, since they were all equal to zero during the break period. To deal with this difficulty, a Stimulus ON vector was included in the matrix, which was equal to one during image presentation and zero during the breaks. In addition, all feature vectors, representing the luminance and arousal scores of the images, were standardized and their value during the breaks was set to their average value. This procedure removed the correlation between the feature vectors and accounted for the overall change in pupil size between breaks and image presentation epochs.

## Results

**Figure 1:**
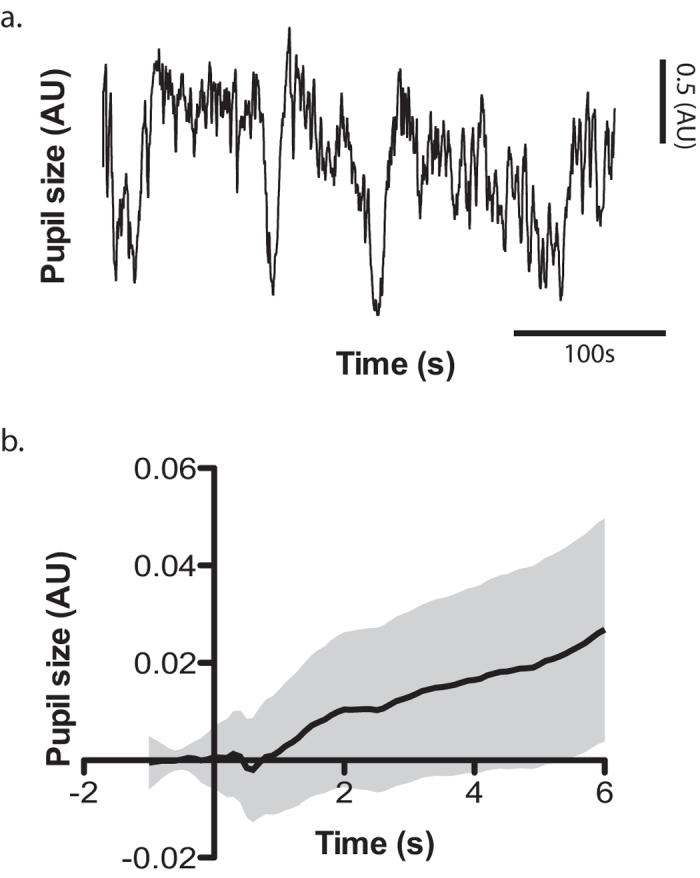
Example pupil trace and baseline-corrected average. (**a**) Pupil trace of one example block illustrating the high-level of low-frequency noise in the data. (**b**) Baseline-corrected average of one example block. The actual pupil response to the image presentation is masked by the large upward slope, which failed to be suppressed by baseline subtraction. The shaded gray area illustrates the 95% confidence interval.

**Figure 2:**
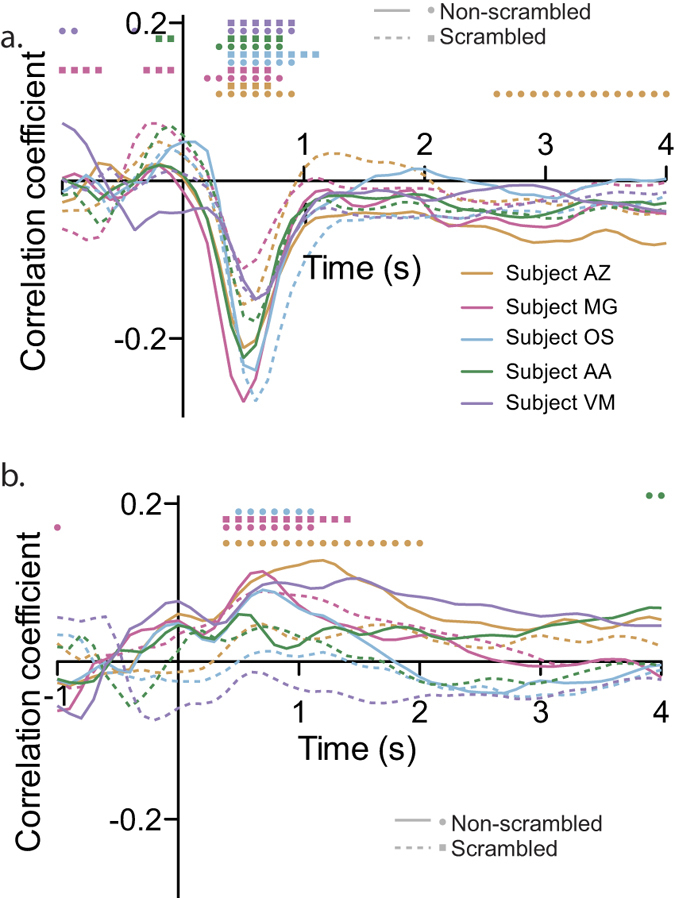
Correlation between the Lab luminance (**a**) and arousal score (**b**) of the affective pictures and the baseline-corrected pupil response as a function of time. Subjects are color-coded, while non-scrambled and scrambled blocks are represented with the solid and dashed lines, respectively. Dots (square for non-scrambled and circles for scrambled blocks) on the top of each panel mark the bins in which the correlation was significant (alpha = 0.05).

I performed first a standard analysis of the pupil data. After baseline correction of the pupil signal recorded during the image-viewing task, strong influence of the low-frequency noise remained (mostly because this method does not correct the slope of the signal; see example subject in Fig. 1). Correlation of this raw baseline-corrected signal with the image luminance was significant in all subjects for both scrambled and non-scrambled images (see Fig. 2a, significant time bins between 200 and 1100 ms, alpha = 0.05). In contrast, and as expected, correlation with arousal score was less consistent, with only 3 subjects showing significant correlations, between 400 and 2000 ms (see Fig. 2b). Since the point of this first analysis was to compare it with the new proposed method, no correction for multiple comparisons was used. Despite this absence of correction, the sensitivity of the method was still inferior to the one obtained with the ARX technique (see below).

**Figure 3:**
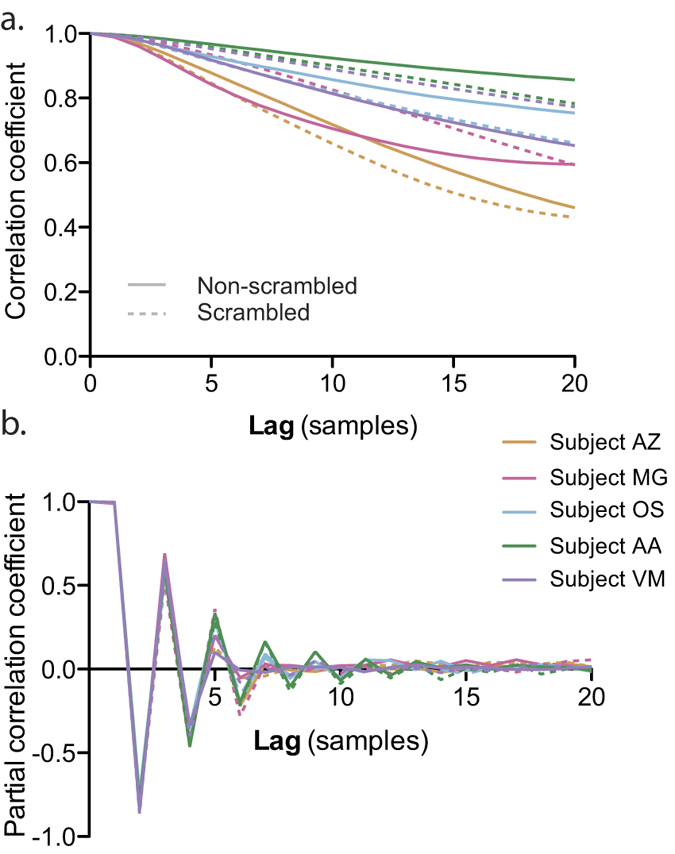
Autocorrelation (**a**) and partial autocorrelation (**b**) of the pupil data. Same conventions as in Fig. 2.

Then, in an attempt to determine which model to use for the pupil time series, autocorrelation (i.e. how much the signal at time t correlates with the signal at time t + lag) and partial autocorrelation functions (i.e. how much the signal at time t correlates with the signal at time t + lag, accounting for the correlations with the signal between time bins t to t + lag-1) were computed. In all 5 subjects, the autocorrelation function decreased slowly, without converging to zero, while the partial autocorrelation function converged after about 10 lags (see Fig. 3). In addition, an augmented Dickey-Fuller test (adftest in matlab) confirmed that all signals were stationary (all p values < 0.05). These results are the signature of a simple autocorrelative process^29^, calling for an autoregressive (AR) model, as opposed to autoregressive moving average (ARMA) or autoregressive integrated moving average (ARIMA) models, which lead to different aucorrelation and partial autocorrelation functions.

The order *p* of the AR model was chosen sessionwise so as to minimize the Akaike Information Criterion and the model was fitted by minimizing the square error of the t + 1 prediction (see Methods). The predicted signal was computed by taking *p* successive values, applying the parameters of the fitted model, and estimating the value at t + 1. The innovation error was the difference between the measured signal and the predicted signal. This innovation error thus represents the information in the signal that is not predicted from earlier values, and is assumed to be normally distributed in the AR model. The distribution of these innovation errors was inspected by means of Q-Q plots and by plotting them as a function of time. They tended to deviate slightly from normality with a negative skewness (−0.6642 ± 0.0977, mean ± SE) and large kurtosis (i.e. heavy tails, 18.0621 ± 5.5236) but showed no systematic drift over time (Spearman correlations with time <0.1). As expected, most of the autocorrelation and partial autocorrelation functions of the innovation error were flat (all below significance threshold), indicating that the AR model accounted successfully for the autocorrelations in the signal. This was confirmed statistically by running a Ljung-Box Q-test for residual autocorrelations. All tests were negative (p values > .1) except for one innovation error signal (scrambled images, subject AA; p = 0.0079), for which autocorrelation and partial autocorrelation coefficients were barely above the significance threshold (0.0338) in time lags 7, 8 and 9. This signal was characterized by a larger blink rate than average (11% of the signal composed of blinks, compared to 6% on average), which may have explained the failure of the AR model to account for all the autocorrelation structure of the data. Overall, these deviations from normality and remaining autocorrelation structure of the innovation error were very small and considered negligible in the context of our particular application.

**Figure 4:**
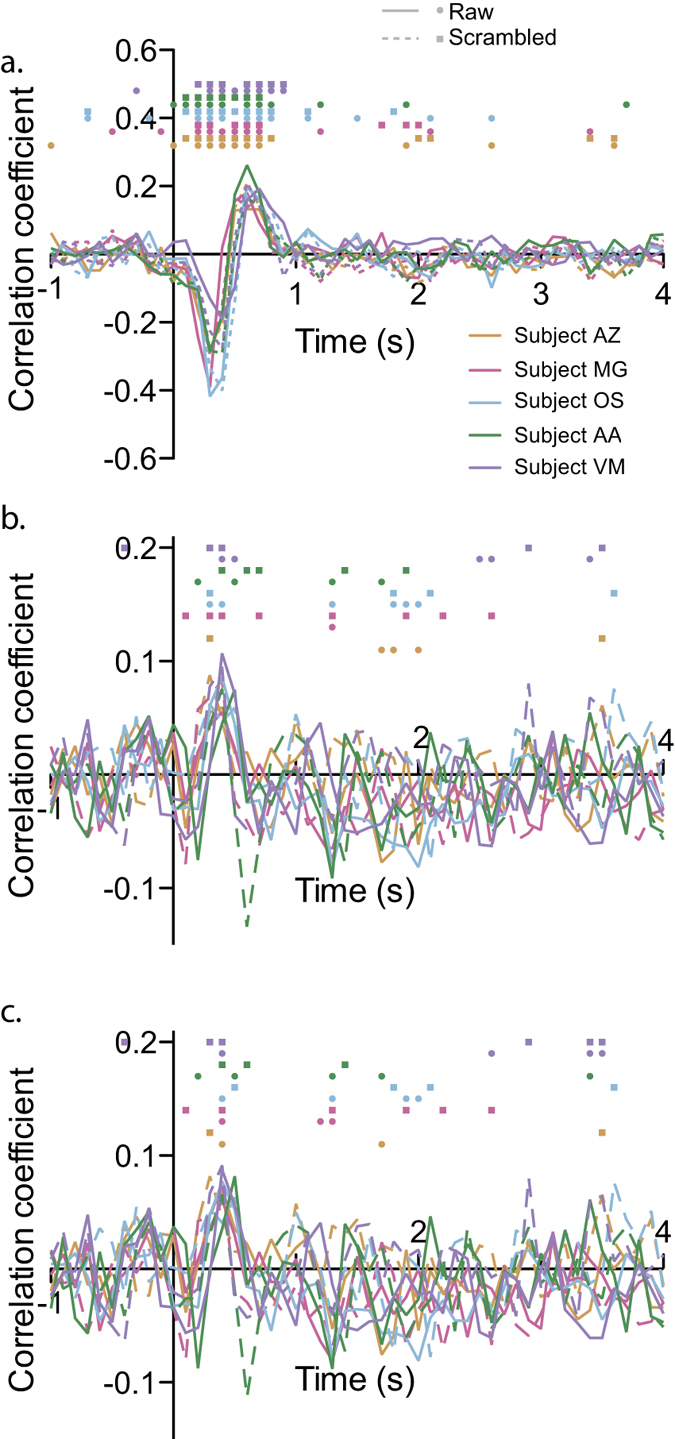
Correlation between the Lab luminance (**a**) and arousal score (**b** and **c**) of the affective pictures and the innovation error of the AR model as a function of time. Same conventions as in Fig. 2. While panel b. shows the correlation with the arousal score, panel c. illustrates the partial correlation, accounting for all the low-level features of the images.

The correlation of the innovation error with the luminance parameter was very consistent across subjects, peaked negatively between 300 and 400 ms after image onset, and was followed by a positive peak, 500 to 800 ms after image onset (see Fig. 4a). The correlation with arousal showed also a consistent positive peak between 300 and 800 ms (see Fig. 4b, all subjects show significant correlations within this time frame). However, it was present even for scrambled images, suggesting the presence of a confounding factor. Since the arousal score correlated with some of the low-level features of the images (significant only for the Lab a value: rho = 0.13, p < 0.001; all other p values > 0.05), partial correlations between pupil response and arousal were performed to account for this putative confound, removing the effect of all the low-level features of the image (Lab luminance, a and b values, image contrast and entropy). However, the results were very similar, and significant correlations with the arousal score were still present for scrambled images (see Fig. 4c). This suggests complex influences of the low-level image features on the pupil response, which failed to be removed by simple partial correlations.

**Figure 5:**
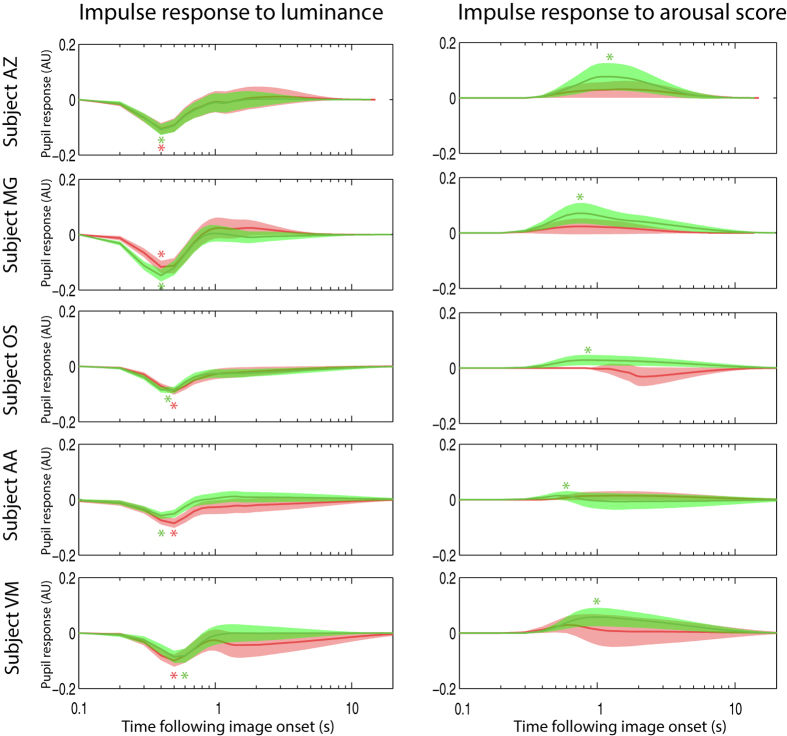
Impulse responses to Lab luminance and arousal scores in all participants. Shaded areas indicate the 99% confidence interval. Green curves correspond to non-scrambled blocks, while red curves correspond to data from the scrambled blocks. Asterisks indicate peak values within the significant bins.

**Figure 6:**
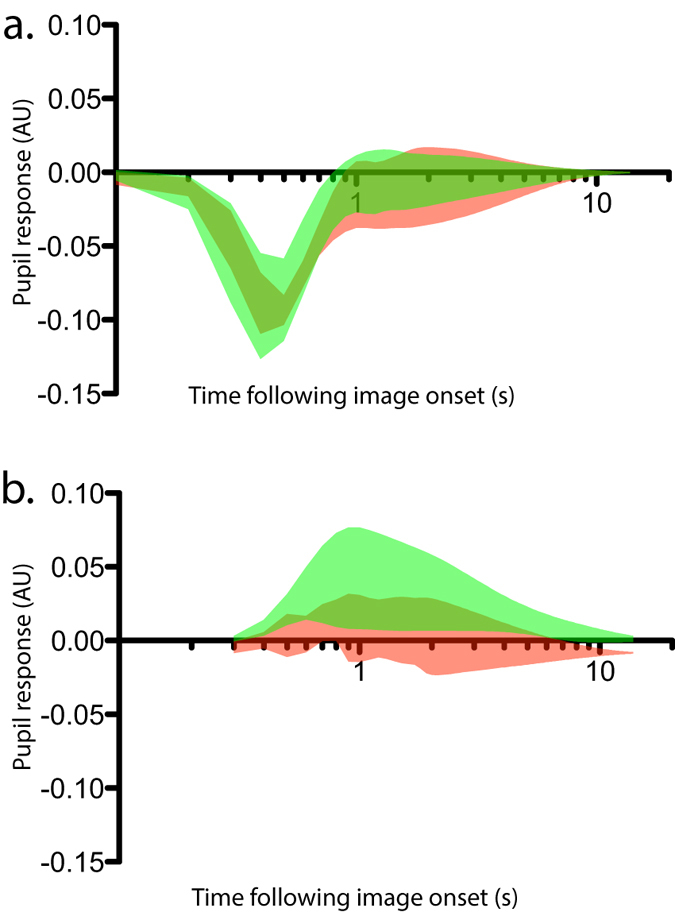
Population averaged impulse responses to Lab luminance (**a**) and arousal score (**b**). Same convention as in Fig. 5.

Finally, exogenous inputs were added to the model. Impulse responses (i.e. the modeled responses of the pupil to an input of value 1 with a duration of 1 sample) with confidence intervals were extracted for each exogenous input, each subject and for both scrambled and non-scrambled images. In all 5 subjects, the pupil showed very clear impulse responses to the luminance of the image, which differed very little within and between subjects (see Fig. 5; note that since impulse responses are issued from the model, they can be displayed with arbitrarily long duration, without interference from subsequent stimuli). In addition, in all subjects, the impulse response to the arousal score was significant (i.e. the confidence interval on Fig. 5 did not cross zero, alpha = 0.01) in blocks during which non-scrambled images were presented, but not in blocks showing scrambled images. From these results, various second-level population analyses could be performed, such as analyses of variance on the peak amplitude of the impulse responses or on their latency. Here, as an example of second-level analysis, I computed the population average of the impulse response, combining the errors in the individual estimates by means of a random effect meta-analysis model^30^ (function averageImpulseResponses in the proposed toolbox). This population average confirmed the clear response to luminance in both scrambled and non-scrambled blocks and the significant response to arousal restricted to non-scrambled blocks (see Fig. 6).

In the pupil size data, eye blinks were linearly interpolated. However, this does not by itself guarantee that blinks did no influence the results, especially if the tagging of the blinks was not perfect, and if the occurrence of those blinks depended on the other regressors (i.e. if they were more likely to occur following images with high luminance and/or high arousal content). In order to determine whether the luminance and arousal scores of the images influenced the probability of the occurrence of the blinks, logistic regressions were performed in each 100-ms time bin of the 2 seconds that followed each image. Despite the absence of correction for multiple comparisons, none of the tests were significant for the luminance regressor (alpha = 0.01), and the tests were significant only in 2 bins, for one subject, in the non-scrambled image presentation session for the arousal regressor (with a slight decrease in blink probability following high arousal images, from 1100 to 1300 ms following image onset). This shows that luminance and arousal had none to minor effect on blink occurrence.

In addition, in order to really ensure that the observed effect of luminance and arousal on the pupil response was not caused by blinks (including in the subject whom showed this weak relation between arousal and blink probability), a blink regressor was added to the ARX model described above, leading to a total of 4 regressors in the model. The pupil was found to react significantly to the blinks in all subjects, showing a strong negative response to blink offset (caused by the global change in luminance which follows eyelid opening). However, accounting for the pupil response to blinks in the ARX model did not change the modeled response to arousal, which still differed significantly from zero in the non-scrambled image presentation data. These results confirm that the pupil response to arousal is not caused by an artifact related to blink occurrence.

**Figure 7:**
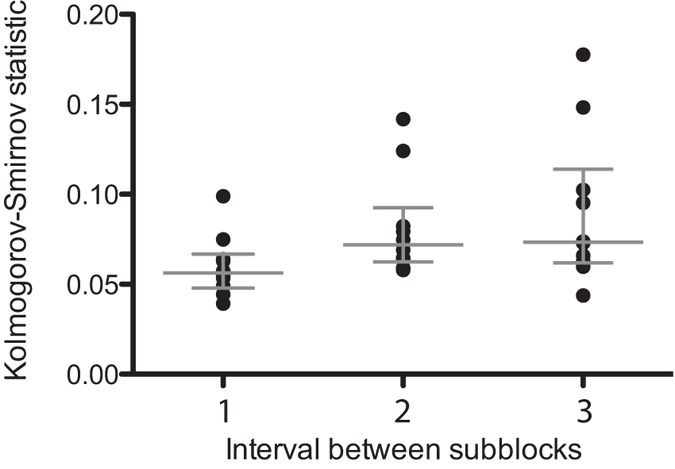
Difference between innovation error distributions (estimated from the Kolmogorov-Smirnov statistic shown on the y-axes) across sub-blocks, as a function of the time interval between sub-blocks. Each circle corresponds to one experimental block (either scrambled or non scrambled) from one subject. Gray lines indicate median and interquartile range.

In order to determine the stability of the ARX model over time, the distributions of the innovation errors were compared (Kolmogorov-Smirnov test) across the four sub-blocks of presentation in each session (corresponding to constant image presentation separated by 7 s breaks). The average Kolmogorov-Smirnov statistic increased as a function of the time distance between the sub-blocks (see Fig. 7; Friedman test: χ^2^ = 12.6, p = 0.0018), indicating that the underlying process, governing the pupil responses and captured partly by the ARX model, changed over time. These changes could be caused by different factors that were not measured in the present study, such as fatigue, sleepiness or mind wandering. This suggests that tracking the parameters of the ARX model over time could potentially be used to isolate different cognitive states of the subjects. This however, will require further experiments.

**Figure 8:**
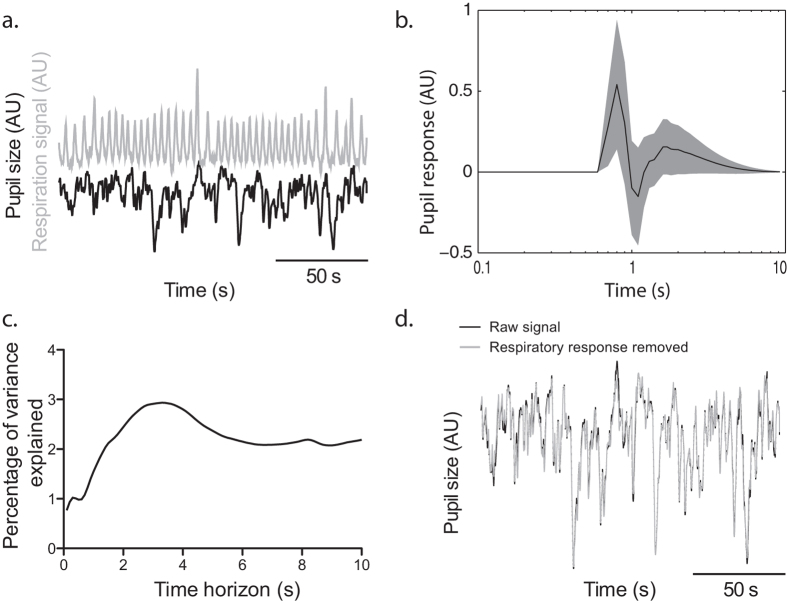
Influence of respiration on the pupil signal in one example subject. (**a**) The pupil size (black trace) and the pressure in the waist cuff (gray trace) were measured concurrently. (**b**) ARX analysis highlighted a significant influence of the respiration signal on the pupil size. The shaded area indicates the 99% confidence interval of the impulse response. (**c**) The percentage of variance explained depended on the time horizon - i.e. how far ahead in time was the model prediction – but topped around 3%. (**d**) The effect of respiration was then removed from the pupil trace, to illustrate the capacity of this approach to clean the signal from unwanted influences.

Finally, the data from the rest recording session was analyzed, with the objective of illustrating the possibility to use continuous physiological signals as inputs in the models (see Fig. 8a). The respiration signal was used to predict pupil variations in one subject. The resulting impulse response differed significantly from zero (see Fig. 8b), even though the percentage of variance of the pupil explained by the respiratory influence was only around 3% (see Fig. 8c). In order to remove the confounding effect of the respiration on the signal, the autoregressive part of the impulse response (the *φ* parameters in the equation described in Methods) was applied on the innovation error from the ARX model. The difference between the raw signal and the signal after removal of the response to the respiration signal is shown in Fig. 8d.

## Discussion

In the present paper, I describe a new approach to analyze pupillometric data that addresses the issue of autocorrelations and low-frequency noise in the pupil signal. This method allowed me to perform subject-by-subject analysis of the pupil response to the luminance and arousal scores of standardized images, presented in rapid succession. This fast pace yielded pupil responses to more than a thousand images in about 6 minutes. In all participants, the pupil reacted significantly to the arousal score of the images, in agreement with earlier findings^31^,^32^,^33^.

Various approaches have been proposed to deal with the issues of pupillometric data analysis. Hoeks & Levelt devised a method based on deconvolution^17^. First, a parametric, canonical impulse response was determined by optimizing the fit between pupillometric data and an input vector representing events of interest, convolved with the impulse response. A fitting procedure was used in which free parameters controlled the shape of an impulse response, which was then convolved with an input vector representing the events of interest. This fitting procedure allowed Hoeks & Levelt to determine the parameters that maximized the fit between the convolved input vector and the actual data. Once these parameters are determined, they can be used to perform the inverse approach, i.e. to determine the input vector by deconvolving the impulse response from the pupillometric data^17^,^18^, or to perform general linear model analysis, similar to the standard univariate analyses used in neuroimaging data^28^, by regressing predicted responses on actual pupillometric data^19^. Despite its merits, this technique has also important limitations, which the present method addresses. First, the present method deals with the variability of the impulse responses in terms of latency and intensity, across individuals and across stimulation features (e.g. luminance versus arousal). This variability highlights the need for adapting the parameters subjectwise, rather than using canonical impulse responses^17^,^18^,^19^. Second, this approach deals automatically with the issue of baseline correction. Because of the typical low-frequency noise inherent to the pupil data, event-aligned pupil traces can have strong upward or downward trends, which are not corrected by baseline correction. Detrending or high-pass filtering the pupil traces are not optimal solutions either, because they require to make assumptions about the frequency of the noise and the frequency of the signal. The ARX model provides thus an elegant solution to this issue.

The present approach is straightforward to implement in any programming language including system identification libraries, such as Matlab, Python or others. Its decisive advantage is most obvious in situations in which the stimuli are presented in rapid succession, the responses are weak or the influence of several features of the stimuli are addressed at the same time. However, in simple experimental designs in which the pupil signal is recorded for several seconds after each stimulus, such that its response can be easily isolated, in which the number of conditions is limited and in which the effect size is large, there is probably no advantage in using such approach, and a standard averaging of the signal, combined with standard hypothesis testing methods could be sufficient. Another major potential advantage of the present method is the possibility to include other physiological signals as inputs to the model. Here, in order to illustrate this possibility, I have modeled the response of the pupil to respiration, but other physiological signals, such as neural signals, could also be used. This could allow researchers to assess how neural activity, such as electroencephalographic signals, local field potentials or single cell recordings can be involved in driving the pupil response^12^. A last possibility for extension of this method is to include multiple outputs in the models, allowing interactions to occur between the signals. This approach is the basis of the Granger causality measures, now popular in neuroimaging analysis^34^. This could allow neural signals to be modeled as outputs, together with the pupil signal, while the inputs would remain limited to actual exogenous events.

In summary, the present paper introduces a novel approach to pupillometric data analysis, which exhibits some decisive advantages over previous methods and opens various possibilities for improvement and extension in the future.

## Additional Information

**How to cite this article**: Zénon, A. Time-domain analysis for extracting fast-paced pupil responses. *Sci. Rep.* **7**, 41484; doi: 10.1038/srep41484 (2017).

**Publisher's note:** Springer Nature remains neutral with regard to jurisdictional claims in published maps and institutional affiliations.

## References

[b1] Joyce D. S., Feigl B., Cao D. & Zele A. J. Temporal characteristics of melanopsin inputs to the human pupil light reflex. Vision Res. 107, 58–66 (2015). 25497360 10.1016/j.visres.2014.12.001 PMC4308541

[b2] Gamlin P. D., Zhang H. & Clarke R. J. Luminance neurons in the pretectal olivary nucleus mediate the pupillary light reflex in the rhesus monkey. Exp Brain Res 106, 169–176 (1995). 8542972 10.1007/BF00241367

[b3] Wilhelm B. J., Wilhelm H., Moro S. & Barbur J. L. Pupil response components: studies in patients with Parinaud’s syndrome. Brain 125, 2296–2307 (2002). 12244086 10.1093/brain/awf232

[b4] Naber M. & Nakayama K. Pupil responses to high-level image content. J Vis 13, 7–7 (2013). 10.1167/13.6.7 23685390

[b5] Mathôt S., van der Linden L., Grainger J. & Vitu F. The pupillary light response reflects eye-movement preparation. J Exp Psychol Hum Percept Perform 41, 28–35 (2015). 25621584 10.1037/a0038653

[b6] Kahneman D. Attention and effort., doi: 10.1167/14.4.1 (Prentice Hall, 1973).

[b7] Zénon A., Sidibé M. & Olivier E. Pupil size variations correlate with physical effort perception. Frontiers in Behavioral Neuroscience 8, 286 (2014). 25202247 10.3389/fnbeh.2014.00286 PMC4142600

[b8] O’Reilly J. X. . Dissociable effects of surprise and model update in parietal and anterior cingulate cortex. Proc. Natl. Acad. Sci. USA. 110, E3660–9 (2013). 23986499 10.1073/pnas.1305373110 PMC3780876

[b9] Preuschoff K., t Hart B. M. & Einhäuser W. Pupil Dilation Signals Surprise: Evidence for Noradrenaline’s Role in Decision Making. Front Neurosci 5 (2011). 10.3389/fnins.2011.00115 PMC3183372 21994487

[b10] Nassar M. R. . Rational regulation of learning dynamics by pupil-linked arousal systems. Nature Neuroscience 15, 1040–1046 (2012). 22660479 10.1038/nn.3130 PMC3386464

[b11] Jepma M. & Nieuwenhuis S. Pupil Diameter Predicts Changes in the Exploration–Exploitation Trade-off: Evidence for the Adaptive Gain Theory. Journal of Cognitive neuroscience 23, 1587–1596 (2011). 20666595 10.1162/jocn.2010.21548

[b12] Joshi S., Li Y., Kalwani R. M. & Gold J. I. Relationships between Pupil Diameter and Neuronal Activity in the Locus Coeruleus, Colliculi, and Cingulate Cortex. Neuron doi: 10.1016/j.neuron.2015.11.028 (2015). PMC4707070 26711118

[b13] Wang C.-A. & Munoz D. P. A circuit for pupil orienting responses: implications for cognitive modulation of pupil size. Current Opinion in Neurobiology 33, 134–140 (2015). 25863645 10.1016/j.conb.2015.03.018

[b14] McGinley M. J., David S. V. & McCormick D. A. Cortical Membrane Potential Signature of Optimal States for Sensory Signal Detection. Neuron 87, 179–192 (2015). 26074005 10.1016/j.neuron.2015.05.038 PMC4631312

[b15] Reimer J. . Pupil fluctuations track fast switching of cortical states during quiet wakefulness. Neuron 84, 355–362 (2014). 25374359 10.1016/j.neuron.2014.09.033 PMC4323337

[b16] Reimer J. . Pupil fluctuations track rapid changes in adrenergic and cholinergic activity in cortex. Nat Commun 7, 13289 (2016). 27824036 10.1038/ncomms13289 PMC5105162

[b17] Hoeks B. & Levelt W. Pupillary dilation as a measure of attention: A quantitative system analysis. Behav Res (1993).

[b18] Wierda S. M., van Rijn H., Taatgen N. A. & Martens S. Pupil dilation deconvolution reveals the dynamics of attention at high temporal resolution. Proc. Natl. Acad. Sci. USA 109, 8456–8460 (2012). 22586101 10.1073/pnas.1201858109 PMC3365158

[b19] de Gee J. W., Knapen T. & Donner T. H. Decision-related pupil dilation reflects upcoming choice and individual bias. Proc. Natl. Acad. Sci. USA 111, E618–25 (2014). 24449874 10.1073/pnas.1317557111 PMC3918830

[b20] Riera J. . fMRI activation maps based on the NN-ARx model. NeuroImage 23, 680–697 (2004). 15488418 10.1016/j.neuroimage.2004.06.039

[b21] Cerutti S., Chiarenza G., Liberati D., Mascellani P. & Pavesi G. A parametric method of identification of single-trial event-related potentials in the brain. IEEE Trans Biomed Eng 35, 701–711 (1988). 3169822 10.1109/10.7271

[b22] Struys M. M. R. F. . Ability of the bispectral index, autoregressive modelling with exogenous input-derived auditory evoked potentials, and predicted propofol concentrations to measure patient responsiveness during anesthesia with propofol and remifentanil. Anesthesiology 99, 802–812 (2003). 14508310 10.1097/00000542-200310000-00010

[b23] Shooshtari P., Mohammadi G., Ardekani B. & Shamsollahi M. Removing ocular artifacts from EEG signals using adaptive filtering and ARMAX modeling. PROCEEDINGS OF WORLD ACADEMY OF SCIENCE, ENGINEERING AND TECHNOLOGY 11 (2006).

[b24] Watson A. B. & Yellott J. I. A unified formula for light-adapted pupil size. J Vis 12, 12 (2012). 10.1167/12.10.12 23012448

[b25] Pamplona V. F. & Oliveira M. M. Photorealistic models for pupil light reflex and iridal pattern deformation. ACM Transactions on Graphics 28 (2009).

[b26] Marchewka A., Zurawski Ł., Jednoróg K. & Grabowska A. The Nencki Affective Picture System (NAPS): introduction to a novel, standardized, wide-range, high-quality, realistic picture database. Behav Res 46, 596–610 (2014). 10.3758/s13428-013-0379-1 PMC4030128 23996831

[b27] Werner S. & Noppeney U. Superadditive responses in superior temporal sulcus predict audiovisual benefits in object categorization. Cerebral Cortex 20, 1829–1842 (2010). 19923200 10.1093/cercor/bhp248

[b28] Friston K. J., Jezzard P. & Turner R. S. Analysis of functional MRI time-series. Hum Brain Mapp 1, 153–171 (2004).

[b29] Box G., Jenkins G. M. & Reinsel G. C. Time Series Analysis: Forecasting and Control. (Prentice Hall, 1994).

[b30] Borenstein M., Hedges L. V., Higgins J. P. T. & Rothstein H. R. A basic introduction to fixed-effect and random-effects models for meta-analysis. Res Synth Methods 1, 97–111 (2010). 26061376 10.1002/jrsm.12

[b31] Bradley M. M., Miccoli L., Escrig M. A. & Lang P. J. The pupil as a measure of emotional arousal and autonomic activation. Psychophysiology 45, 602–607 (2008). 18282202 10.1111/j.1469-8986.2008.00654.x PMC3612940

[b32] Partala T. & Surakka V. Pupil size variation as an indication of affective processing. International Journal of Human-Computer Studies 59, 185–198 (2003).

[b33] van Steenbergen H., Band G. P. H. & Hommel B. Threat but not arousal narrows attention: evidence from pupil dilation and saccade control. Front Psychol 2, 281 (2011). 22059081 10.3389/fpsyg.2011.00281 PMC3204575

[b34] Rogers B. P., Katwal S. B., Morgan V. L., Asplund C. L. & Gore J. C. Functional MRI and multivariate autoregressive models. Magnetic resonance Imaging 28, 1058–1065 (2010). 20444566 10.1016/j.mri.2010.03.002 PMC2940955

